# Severe traumatic brain injury and hypotension is a frequent and lethal combination in multiple trauma patients in mountain areas – an analysis of the prospective international Alpine Trauma Registry

**DOI:** 10.1186/s13049-021-00879-1

**Published:** 2021-04-30

**Authors:** Simon Rauch, Matilde Marzolo, Tomas Dal Cappello, Mathias Ströhle, Peter Mair, Urs Pietsch, Hermann Brugger, Giacomo Strapazzon, the IATR study group, Martin Palma, Lukas Gasteiger, Marianna Zatelli, Elke Frohn, Erika Noe, Stefanie Ziegler, Andreas Frasnelli, Samuel Haupt, Marc Kaufmann, Elisabeth Gruber, Nicole Ritsch, Rita Haller, Wolfgang Lunz, Katharina Grasegger

**Affiliations:** 1 Eurac Research, Institute of Mountain Emergency Medicine, Bolzano, Italy; 2 Department of Anaesthesiology and Intensive Care, Hospital of Merano, Merano, Italy; 3 Department of Anaesthesiology and Intensive Care, University Hospital Innsbruck, Innsbruck, Austria; 4 Department of Anaesthesiology and Intensive Care, St. Gallen Hospital, St. Gallen, Switzerland; 5 Swiss Air Rescue Rega, Zürich, Switzerland

**Keywords:** Trauma, Traumatic brain injury, Hypotension, Shock, Mountain rescue

## Abstract

**Background:**

Hypotension is associated with worse outcome in patients with traumatic brain injury (TBI) and maintaining a systolic blood pressure (SBP) ≥110 mmHg is recommended. The aim of this study was to assess the incidence of TBI in patients suffering multiple trauma in mountain areas; to describe associated factors, treatment and outcome compared to non-hypotensive patients with TBI and patients without TBI; and to evaluate pre-hospital variables to predict admission hypotension.

**Methods:**

Data from the prospective International Alpine Trauma Registry including mountain multiple trauma patients (ISS ≥ 16) collected between 2010 and 2019 were analysed. Patients were divided into three groups: 1) TBI with hypotension, 2) TBI without hypotension and 3) no TBI. TBI was defined as Abbreviated Injury Scale (AIS) of the head/neck ≥3 and hypotension as SBP < 110 mmHg on hospital arrival.

**Results:**

A total of 287 patients were included. Fifty (17%) had TBI and hypotension, 92 (32%) suffered TBI without hypotension and 145 (51%) patients did not have TBI. Patients in group 1 were more severely injured (mean ISS 43.1 ± 17.4 vs 33.3 ± 15.3 vs 26.2 ± 18.1 for group 1 vs 2 vs 3, respectively, *p* < 0.001). Mean SBP on hospital arrival was 83.1 ± 12.9 vs 132.5 ± 19.4 vs 119.4 ± 25.8 mmHg (*p* < 0.001) despite patients in group 1 received more fluids. Patients in group 1 had higher INR, lower haemoglobin and lower base excess (*p* < 0.001). More than one third of patients in group 1 and 2 were hypothermic (body temperature < 35 °C) on hospital arrival while the rate of admission hypothermia was low in patients without TBI (41% vs 35% vs 21%, for group 1 vs 2 vs 3, *p* = 0.029). The rate of hypothermia on hospital arrival was different between the groups (*p* = 0.029). Patients in group 1 had the highest mortality (24% vs 10% vs 1%, *p* < 0.001).

**Conclusion:**

Multiple trauma in the mountains goes along with severe TBI in almost 50%. One third of patients with TBI is hypotensive on hospital arrival and this is associated with a worse outcome. No single variable or set of variables easily obtainable at scene was able to predict admission hypotension in TBI patients.

**Supplementary Information:**

The online version contains supplementary material available at 10.1186/s13049-021-00879-1.

## Background

Traumatic Brain Injury (TBI) is the single most important cause of death from traumatic injury and represents a major cause of long-term disability among survivors [1]. Optimal pre-hospital management of patients with TBI can contribute to a favourable outcome and primarily focusses on the prevention of secondary injuries [2]. Within secondary injuries, hypotension plays a major role as its depth and duration have been associated with increased mortality and worse functional outcome [3–5]. Data on the prevalence of hypotension as a preventable cause of secondary injury is limited and available numbers are widely variable. A multicentre study from Switzerland found a rate of pre-hospital hypotension (defined as systolic blood pressure, SBP ≤90 mmHg) of 4.1% in multiple trauma patients with TBI [6]. In a prospective study in the US, 24% of patients with TBI had hypotension (SBP ≤90 mmHg) in the emergency department of a level-I trauma centre caring for urban multiple trauma patients [4]. While in these studies hypotension was defined as SBP ≤90 mmHg, current recommendations advise that patients with TBI should be considered hypotensive for SBP < 110 mmHg [2, 7]. When using a cut-off for SBP of 110 mmHg, the frequency of hypotension occurring in the initial phase of resuscitation in TBI patients could be expected to be even higher than reported so far.

Avoiding hypotension can be challenging in the pre-hospital setting. Pre-hospital rescue missions commonly feature prolonged times and patients often have critically impaired vital functions [8–10]. Hypotension in multiple trauma patients with TBI in mountain areas could therefore be even more common than in urban areas.

There is a lack of knowledge about the prevalence of hypotension in multiple trauma patients with TBI and the factors associated with it in mountain areas. The aim of the present study was to assess the incidence of TBI in patients suffering multiple trauma in mountain areas; to describe associated factors, treatment and outcome of these patients compared to non-hypotensive patients with TBI and patients without TBI; and to evaluate pre-hospital variables able to predict admission hypotension in TBI patients.

## Methods

Out-of-hospital and in-hospital data collected from trauma patients included in the International Alpine Trauma Register (IATR) (https://www.mountain-registries.org) [11] between December 2010 and October 2019 were analysed. The IATR is an international platform for the prospective collection and storage of data relating to severe trauma patients (Injury Severity Score, ISS ≥16) in mountain areas, that are not readily accessible by wheeled emergency vehicles. All trauma patients aged 16–80 years with an ISS ≥16 are included in the analysis and are called “multiple trauma patients” in this article. The registry is hosted in Bolzano (Italy) [11], and data are collected in North Tyrol (Austria), South Tyrol (Italy), Aosta region (Italy) and Chur area (Switzerland). Patients already in cardiac arrest upon arrival of the rescue team, burn patients (if the burn represented the predominant injury), and drowned patients are excluded from the IATR. In North Tyrol (Austria), patients who suffered accidents on prepared ski slopes are excluded. Only patients with vital signs at hospital admission were included in the current study. Data collection in the IATR is based on the Utstein-Style [12], which requires comprehensive data collection on multiple parameters. These parameters include accident (type of outdoor activity, mechanism of injury) and mission characteristics (technical difficulty of the terrain, terrestrial rescue, air rescue or combined rescue) and timing (time of accident, time of emergency call, time of arrival of the first rescue team, time of hospital admission). Medical data collected include: i) vital signs at the scene (i.e. SBP, respiratory rate, Glasgow Coma Scale (GCS) and body temperature); ii) out-of-hospital Advance Trauma Life Support (ATLS) interventions (i.e. endotracheal intubation, intravenous cannulation, fluid and drug administration); iii) ISS and Abbreviated Injury Scale (AIS) based on in-hospital diagnosis; iv) vital signs and laboratory data on admission (i.e. haemoglobin, INR, base excess, body temperature), plus out-of-hospital and in-hospital mortality rates.

### Statistical analysis

Patients were classified into three groups based on clinical characteristics: group 1 included multiple trauma patients with TBI (defined as AIS ≥3 in the head/neck ISS body region) and hypotension defined as SBP ≤110 mmHg [2, 7] on hospital arrival; group 2 included multiple trauma patients with TBI but without hypotension on hospital arrival; group 3 included multiple trauma patients without TBI. Frequencies between the three groups were compared by means of Pearson’s chi-squared test, while for the two-group comparison Fisher’s exact test was used. Mean values between the three groups were compared by means of ANOVA or Kruskal-Wallis test as appropriate, while for the two-group comparison the independent samples *t*-test or Mann-Whitney *U* test was used as appropriate. Paired samples *t*-tests were performed to compare pre-hospital SBP and SBP on hospital arrival. The Holm-Bonferroni method was used to correct *p*-values for multiple comparisons.

Classification trees [13, 14] were performed to predict the three groups defined above, i.e. classification trees predicted whether patients will have TBI or not, and in case they had TBI, whether they will have admission hypotension or not. Unlike logistic and linear regression, classification trees do not develop a prediction equation. Instead, data are partitioned along the predictor axes into subsets with homogeneous values of the dependent variable, a process represented by a decision tree that can be used to make predictions from new observations [13]. We assessed the following pre-hospital variables: gender, age, treatment free interval, mechanism of injury (avalanche, collision, fall, other), type of injury (blunt, penetrating), ISS, GCS, SBP, quantity of crystalloid, hypertonic and (hyper) oncotic fluids, use of vasopressors, tracheal intubation and AIS scores (of the following ISS body regions: face, chest, abdomen, extremity and external). The lengths of the branches of the classification trees is proportional to the discriminant ability of the variable (the longer the more discriminant). Cross-validation was used to evaluate the classification trees, i.e. a classification tree was created using 75% of cases (training set), randomly chosen, and its predicting ability was then evaluated on the remaining 25% of cases (testing set), calculating sensitivity and specificity. This procedure was repeated ten times and so ten classification trees were created, each generated by a different training set composed of 75% of cases selected randomly. SPSS version 25 statistical software (IBM Corp., Armonk, NY) was used. The classification trees were built by means of the library rpart of R version 3.4.1 [14, 15]. Tests were two-sided and *p* < 0.05 was considered statistically significant. Values are reported as mean ± standard deviation unless stated otherwise.

## Results

During the study period, a total of 308 cases were recorded in the IATR. Of them, 21 were excluded (17 patients had missing values for either pre- or in-hospital SBP and four patients suffered cardiac arrest during transport), and 287 patients were finally included in the analysis. A total of 142 patients had TBI, of them 50 patients (17%) had TBI and hypotension on hospital arrival *(group 1)*, 92 (32%) suffered TBI but were not hypotensive on hospital arrival *(group 2)*; 145 (51%) patients did not have TBI *(group 3)*.

Demographics, type of activity leading to the accident and mechanism of injury are shown in Table 1. A difference between groups was found for gender (higher proportion of females in group 1 in comparison to group 3, *p* = 0.033) and for hiking (type of activity before the accident, performed more frequently in group 1 than in group 3, *p* = 0.002).

**Table 1:** Demographics, type of activity leading to the accident and mechanism of injury, subdivided by the three subgroups

	TBI with hypotension (group 1) ***n*** = 50	TBI without hypotension (group 2) ***n*** = 92	No TBI (group 3) ***n*** = 145	***p***-value
***Demographics***				
Age, years, mean ± SD	46.4 ± 19.2	42.4 ± 18.5	44.5 ± 17.2	0.452
Female sex, n (%)	16 (32%)	20 (22%)	21 (14%)	0.024
***Type of activity***				
Aviation, n (%)	1 (2%)	4 (4%)	15 (10%)	0.066
Climbing, n (%)	6 (12)	15 (16%)	16 (11%)	0.478
Hiking, n (%)	22 (44%)	24 (26%)	26 (18%)	0.001
Mountain biking, n (%)	0 (0%)	2 (2%)	10 (7%)	0.056
Ski / snowboard, n (%)	11 (22%)	33 (36%)	53 (37%)	0.141
Sledging, n (%)	2 (4%)	2 (2%)	3 (2%)	0.738
Other, n (%)	8 (16%)	11 (12%)	21 (15%)	0.786
***Mechanism of injury***				
Avalanche, n (%)	1 (2%)	4 (4%)	8 (6%)	0.579
Collision with object / other person, n (%)	11 (22%)	29 (32%)	27 (19%)	0.087
Fall, n (%)	37 (76%)	59 (64%)	105 (74%)	0.203
Other, n (%)	0 (0%)	0 (0%)	2 (1%)	0.368

Tests performed were Pearson’s chi-squared tests, except for age ANOVA

*TBI* traumatic brain injury

Pre-hospital times, SBP, Glasgow Coma Scale (GCS) and selected pre-hospital therapeutic interventions, subdivided by the three groups, are shown in Table 2.

**Table 2:** Pre-hospital times, Glasgow Coma Scale (GCS), systolic blood pressure (SBP) and selected pre-hospital therapeutic interventions subdivided by the three groups

	TBI with hypotension (group 1) ***n*** = 50	TBI without hypotension (group 2) ***n*** = 92	No TBI (group 3) ***n*** = 145	***p***-value
***Pre-hospital times***				
Treatment free interval, min, median (range)	27.5 (3–130)	16 (2–1001)	20 (1–469)	0.239
Total pre-hospital time, min, median (range)	82 (30–1560)	88.5 (20–1047)	80 (13–1274)	0.137
***Level of pre-hospital care***				
No care, n (%)	0 (0%)	5 (5%)	9 (6%)	0.195
Basic life support, n (%)	5 (10%)	12 (13%)	36 (26%)	0.014
Advanced life support, n (%)	45 (90%)	73 (80%)	93 (66%)	0.001
***Pre-hospital SBP and GCS***				
Pre-hospital SBP, mmHg, mean ± SD	103.0 ± 26.2	117.8 ± 26.0	116.4 ± 23.3	0.012
Pre-hospital GCS, mean ± SD	8.5 ± 4.3	9.6 ± 4.3	14.3 ± 1.7	< 0.001
Pre-hospital GCS ≤ 8, n (%)	27 (54%)	39 (43%)	2 (1%)	< 0.001
***Selected pre-hospital therapeutic interventions***				
Crystalloid fluids, ml, mean ± SD	758 ± 339	587 ± 381	580 ± 413	0.011
Vasopressor therapy, n (%)	4 (12%)	0 (0%)	2 (2%)	0.005
Intubation, n (%)	28 (56%)	42 (46%)	19 (13%)	< 0.001
Intubation, n (%) in patients with GCS ≤ 8	24 (89%)	35 (90%)	2 (100%)	0.883

Tests performed were Pearson’s chi-squared tests, except for treatment free interval, total pre-hospital time and crystalloid fluids Kruskal-Wallis tests and for pre-hospital SBP ANOVA

*TBI* traumatic brain injury

Patients with TBI and hypotension (group 1) were more severely injured with a mean ISS of 43.1 ± 17.4 compared to 33.3 ± 15.3 (*p* = 0.001) and 26.2 ± 18.1 (*p* < 0.001) in patients of group 2 and 3, respectively. The higher ISS in groups 1 and 2 compared to group 3 was mainly due to a higher AIS in the head/neck ISS body region (Table 3). Neither the total pre-hospital time nor the treatment free interval differed between the three groups.

**Table 3:** Glasgow Coma Scale (GCS), Abbreviated Injury Scale (AIS) divided by body region and laboratory parameters on hospital arrival

	TBI with hypotension (***n*** = 50)	TBI without hypotension (***n*** = 92)	No TBI (***n*** = 145)	***p***-value
***GCS on hospital arrival***	7.5 ± 5.1	8.6 ± 5.3	13.6 ± 3.4	< 0.001
***ISS body region***				
Head/neck, AIS	4.2 ± 1.0	4.0 ± 0.9	1.7 ± 0.5	< 0.001
Face, AIS	3.7 ± 1.2	3.1 ± 0.7	2.2 ± 1.0	< 0.001
Thorax, AIS	3.8 ± 0.8	3.4 ± 1.0	3.5 ± 1.0	0.169
Abdomen, AIS	3.4 ± 1.0	3.2 ± 0.7	3.4 ± 1.0	0.743
Extremities, AIS	3.6 ± 1.0	3.1 ± 0.8	3.4 ± 1.3	0.135
External, AIS	3.0^a^	3.3 ± 0.9	2.7 ± 1.0	0.384
***Selected laboratory values on hospital admission***				
Hemoglobin, g/dl	10.8 ± 2.8	12.7 ± 1.9	12.9 ± 2.2	*<* 0.001
INR	1.6 ± 0.7	1.2 ± 0.2	1.2 ± 0.3	*<* 0.001
aPTT, sec	45.5 ± 39.6	28.2 ± 5.6	28.4 ± 9.3	< 0.001
Base excess	−5.3 ± 6.7	−3.4 ± 4.2	−0.7 ± 7.7	*<* 0.001

Values are reported as mean ± standard deviation. Tests performed were ANOVAs, except for GCS and AIS head and neck Kruskal-Wallis test

*aPTT* activated partial thromboplastin time, *INR* international normalized ratio, *TBI* traumatic brain injury

^a^only one case

**Fig. 1:**
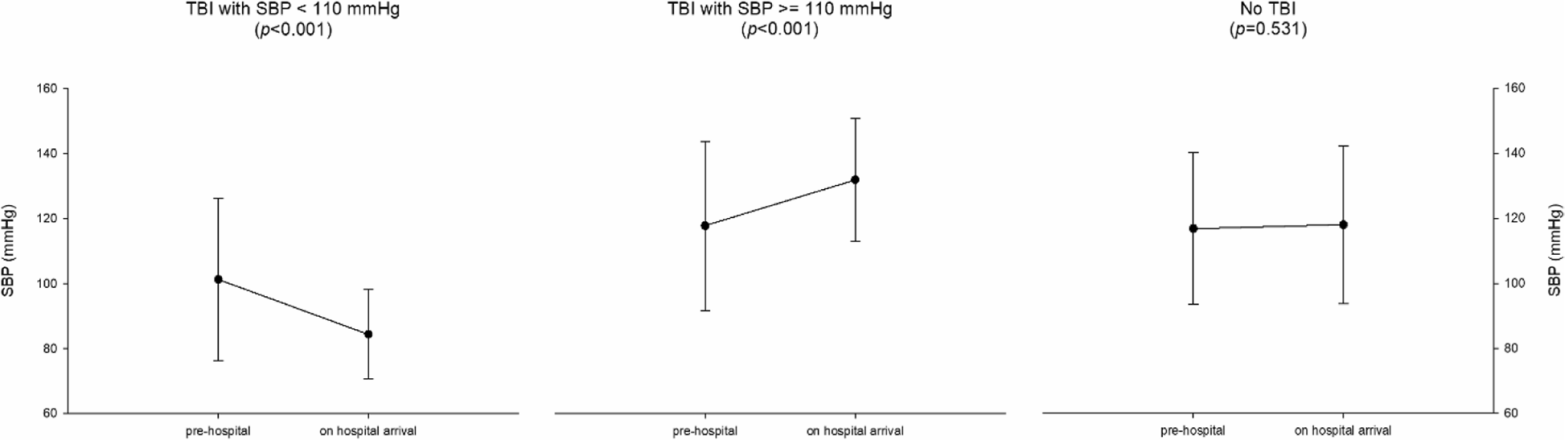
Systolic blood pressure (SBP) change from pre-hospital to on hospital arrival, subdivided by the three groups. Tests performed were paired samples *t*-tests. Error bars represent standard deviation. TBI, traumatic brain injury

Mean SBP on hospital arrival was 83.1 ± 12.9 in group 1 vs 132.5 ± 19.4 in group 2 vs 119.4 ± 25.8 mmHg in group 3 (*p* < 0.001). As depicted in Fig. 1, SBP decreased during the pre-hospital phase in group 1 (relative difference between SBP on scene and SBP on admission = − 12% ± 23%; *p* < 0.001), while it increased in group 2 and remained stable in group 3 (+ 17% ± 30% with *p* < 0.001 and + 2% ± 16% with *p* = 0.531, respectively).

Patients in group 1 received more crystalloid fluids during the pre-hospital treatment as compared to patients in group 2 and 3 (*p* = 0.018 and *p* = 0.012, respectively) (Table 2). On hospital arrival, patients in group 1 as compared to patients in group 2 and 3 had higher INR (*p* = 0.002 and *p* = 0.001, respectively), lower haemoglobin values (*p* < 0.001 and *p* < 0.001, respectively) and lower base excess (*p* = 0.070 and *p* = 0.001, respectively) (Table 3).

Core body temperature on hospital arrival was recorded for 207 patients (for 54 patients, information on the method of body temperature measurement was available: 42 tympanic, 9 bladder, 1 rectal, 1 esophageal, 1 skin/axillary). We found a significant difference in the rate of hypothermia (defined as core body temperature < 35 °C) on hospital arrival between the three groups (41% vs 35% vs 21%, for group 1 vs 2 vs 3, *p* = 0.029). Patients in group 1 had a mortality rate of 24% compared to 10% and 1% in group 2 and 3, respectively (*p* < 0.001). Mortality stratified by categories of SBP values on hospital arrival for patients with TBI (i.e. only group 1 and 2) is shown in Table 4.

**Table 4:** Mortality rate per level of systolic blood pressure (SBP) on hospital arrival, only patients with traumatic brain injury, i.e. group 1 and 2

SBP on hospital arrival	Died in hospital, n (%)
< 90 mmHg, n (%)	6 (25%)
90–99 mmHg, n (%)	3 (30%)
100–109 mmHg, n (%)	1 (13%)
110–119 mmHg, n (%)	4 (25%)
≥ 120 mmHg, n (%)	5 (7%)

(*p* = 0.072, Pearson’s chi-squared test)

There was no difference in the rate of pre-hospital intubation between TBI patients with and without admission hypotension (i.e. group 1 vs 2, 56% vs 46%, *p* = 0.292)*.* In Table 5, patients with TBI and admission hypotension (i.e. patients in group 1) are subdivided into patients who were intubated on scene and patients who were not, and different parameters are compared between the two groups. No difference between the two subgroups was found regarding SBP on scene and on admission, pre-hospital fluid and vasopressor therapy, ISS, AIS of the different body regions, haemoglobin, coagulation parameters or base excess. Only the GCS was lower both on scene and on hospital admission in TBI patients who were intubated on scene.

**Table 5:** Demographics, pre-hospital time, systolic blood pressure (SBP; pre-hospital and on admission), Glasgow Coma Scale (GCS; pre-hospital and on admission), selected pre-hospital therapeutic interventions, Injury Severity Score (ISS), Abbreviated Injury Scale (AIS) and selected laboratory values of patients with TBI and hypotension on hospital admission (group1), subdivided between patients intubated and not intubated on scene

Parameter	TBI with hypotension (***n*** = 50) Pre-hospital intubation		
	Yes	No	***p***-value
Age, years, mean ± SD	42.7 ± 19.6	49.4 ± 18.6	0.243
Female sex, n (%)	7 (32%)	9 (32%)	1.000
Pre-hospital SBP, mmHg, mean ± SD	102.0 ± 22.8	103.8 ± 29.3	0.844
SBP on hospital arrival, mmHg, mean ± SD	81.1 ± 12.2	84.8 ± 13.4	0.325
Pre-hospital GCS, mean ± SD	5.8 ± 3.	11.8 ± 3.2	< 0.001
GCS on hospital arrival, mean ± SD	3.3 ± 1.3	12.0 ± 3.5	< 0.001
Crystalloid fluids, ml, mean ± SD	636 ± 303	821 ± 346	0.194
Vasopressor therapy, n (%)	1 (7%)	3 (16%)	0.613
Total pre-hospital time, min, median (range)	82 (33–221)	82.5 (30–1560)	0.881
ISS, mean ± SD	41.1 ± 13.3	44.6 ± 20.2	0.930
AIS head and neck	3.9 ± 0.9	4.5 ± 1.1	0.054
AIS face	2.5 ± 0.7	4.0 ± 1.2	0.133
AIS thorax	3.8 ± 0.8	3.7 ± 0.9	0.846
AIS abdomen	3.2 ± 0.9	3.6 ± 1.1	0.467
AIS extremities	3.7 ± 0.9	3.6 ± 1.1	0.752
AIS external	3^a^	–	–
Hemoglobin, g/dl	11.3 ± 2.7	10.4 ± 2.8	0.221
INR	1.4 ± 0.6	1.7 ± 0.7	0.254
aPTT, sec	34.3 ± 21.6	54.7 ± 48.6	0.135
Base excess	−6.3 ± 5.1	−4.6 ± 7.7	0.377

Tests performed were independent samples *t*-tests, except for pre-hospital GCS and GCS on hospital arrival, crystalloid fluids, total pre-hospital time, ISS and AIS head/neck, for which Mann-Whitney *U* test was used and for vasopressor therapy and female sex, for which Fisher’s exact test was applied

*INR* international normalized ratio

^a^only one case, *aPTT* activated partial thromboplastin time

### Classification trees

**Fig. 2:**
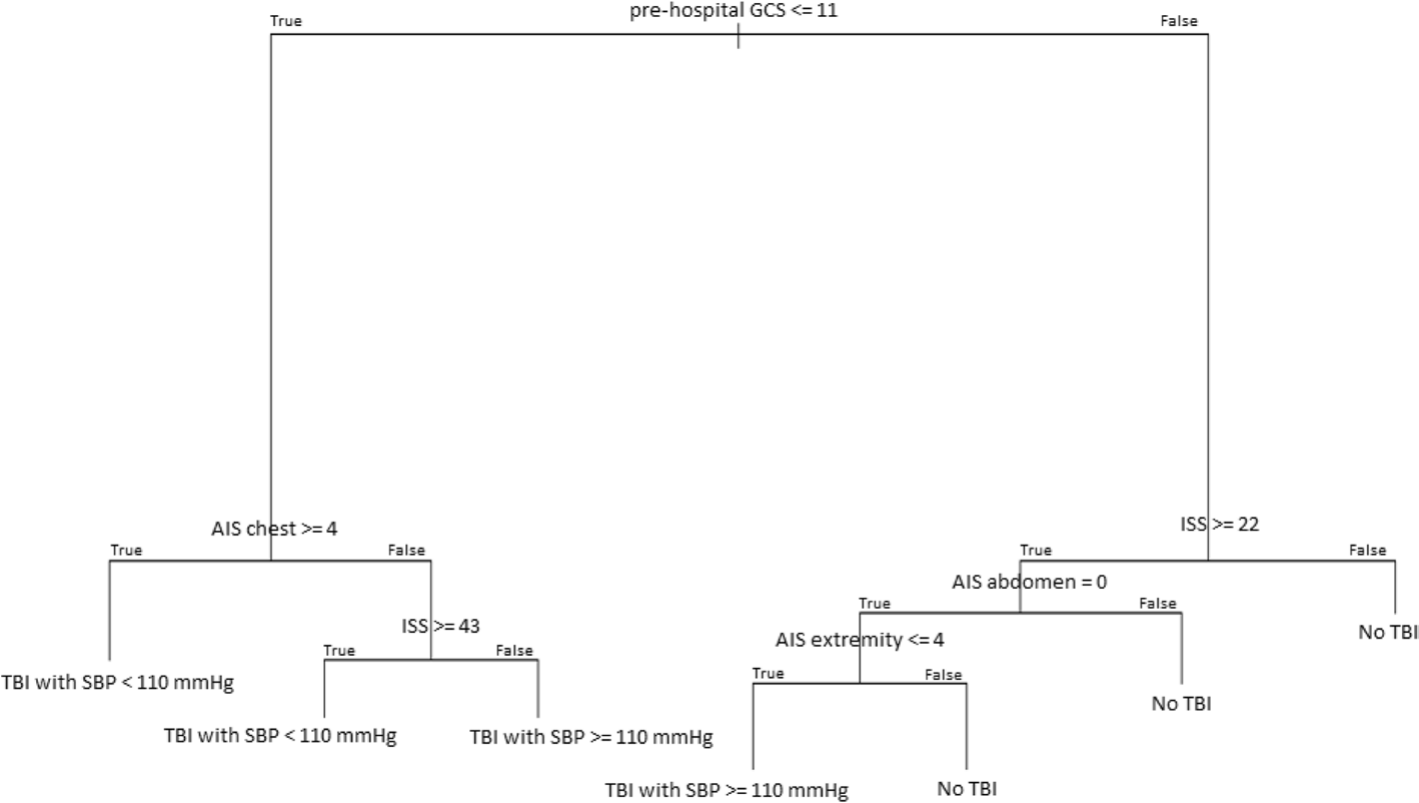
Classification tree to predict traumatic brain injury (TBI) with and without hypotension and no TBI. AIS, Abbreviated Injury Scale; GCS, Glasgow Coma Scale; ISS, Injury Severity Score; SBP, systolic blood pressure

One of the ten classification trees is shown as an example in Fig. 2. The mean sensitivity of the ten classification trees (applying them to the testing sets) was 38% [95% confidence interval (CI) 23%–54%] for TBI with admission hypotension and 54% (95% CI 41%–66%) for TBI without admission hypotension; specificity to detect patients without TBI was 85% (95% CI 82%–88%). The GCS was the only variable selected in all ten classification trees and a cut-off of 11 resulted to be the major discriminant between patients with and without TBI (first node of the tree; high discriminant ability reflected by the long branch). For further discrimination between TBI patients with or without admission hypotension, variable selection by the classification trees was inconsistent (supplemental Table 1); following GCS, the most frequently selected variables were ISS, AIS abdomen and AIS extremities.

## Discussion

We found that half of the patients suffering severe multiple trauma in the mountains had concomitant TBI. One third of them was hypotensive with SBP ≤110 mmHg on hospital arrival, despite they received more fluids pre-hospitally than multiple trauma patients without admission hypotension. Hypotensive multiple trauma patients with TBI were more severely injured, had a more severe coagulopathy, lower haemoglobin levels and lower base excess values; body temperature was lower at hospital arrival. Hypotensive patients with TBI had the highest mortality rate compared to non-hypotensive multiple trauma patients with TBI and multiple trauma patients without TBI. No single variable or set of variables could reliably predict admission hypotension in multiple trauma patients with TBI.

Outdoor activities in the mountains have gained popularity over recent years [16]. In parallel, the number of rescue missions in mountainous environments has increased [17] and the proportion of potentially life threatening injuries has notably risen [8, 9, 18]. Data on TBI in severely injured trauma patients in mountain areas is scarce. We found an incidence of TBI of about 50% among mountain multiple trauma patients, which is comparable to the rate of TBI among severely injured patients in urban or suburban areas [19]. The major cause leading to TBI in mountain accidents was fall during hiking.

Hypotension is common in severely injured patients [8, 9] and is tolerated to a certain degree in order to avoid the adverse effects of early and high-dose fluid resuscitation [20]. This concept of “permissive hypotension” is contraindicated in patients with TBI [2] because the maintenance of an adequate cerebral perfusion is essential to ensure tissue oxygenation of the injured central nervous system and to avoid secondary brain injury [3–5, 21–23]. The current guidelines of the Brain Trauma Foundation recommend maintaining SBP at ≥100 mmHg for patients 50 to 69 years old and at ≥110 mmHg for patients 15 to 49 or over 70 years old [2]. We found that one third of multiple trauma patients rescued in mountainous environment with TBI had SBP ≤110 mmHg on hospital arrival, in agreement with published data on urban trauma patients [4, 6]. These patients (group 1) had a marked drop of their SBP during the pre-hospital phase from a mean SBP of about 100 mmHg on scene to about 80 mmHg on hospital arrival, even though these patients received more aggressive fluid resuscitation as compared to the other groups of trauma patients. This might be due to several reasons. First, patients with TBI and admission hypotension were more severely injured and had a higher mean ISS compared to the other groups. We suspect that these patients had more severe traumatic bleeding, as they had lower haemoglobin levels, lower base excess values and more severe coagulopathy on hospital arrival, and hemorrhage is the most frequent cause of hypotension in trauma patients [24]. Yet, the higher ISS was mainly due to a higher AIS of the head/neck body region, where severe traumatic bleeding leading to hypotension is relatively uncommon. Second, the drop of SBP during the pre-hospital phase could indicate that the treatment performed on scene was either insufficient or that no other treatment was feasible. Stopping the bleeding whenever possible and the cautious administration of fluids and possibly vasopressors are the main options to maintain or augment blood pressure and tissue perfusion [25, 26]. The uncritical administration of fluids can have detrimental effects and lead to or worsen hypothermia, dilution coagulopathy and trauma induced coagulopathy, particularly when administered prehospitally in a cold environment. Therefore, a restricted volume replacement strategy to restore cardiac preload and achieve target blood pressure until bleeding can be controlled is recommended [25, 26]. Although in our study patients with TBI and admission hypotension received significantly more fluids compared to non-hypotensive patients with TBI and patients without TBI, the fluid administration in all three patient groups was restrictive with volumes of far less than 1 l. Vasopressors were seldomly administered. The administration of vasopressors to maintain a sufficient cerebral perfusion pressure could play an important role in the treatment of multiple trauma patients with TBI, in particular in mountain rescue operations with prolonged pre-hospital times. Vasopressors allow to maintain target blood pressure and at the same time to limit the amount of fluid and the negative consequences associated with an excessive volume therapy [27]. However, it remains unclear how to attain the best balance between volume resuscitation and vasopressor administration in order to achieve an adequate cerebral perfusion pressure [28–31]. Current guidelines recommend the administration of vasopressors in addition to fluids to maintain target SBP in the presence of life-threatening hypotension [25, 26]. Recent studies on the use of vasopressin in trauma patients with hemorrhagic shock are promising [32, 33] but additional research is necessary to determine effect on morbidity or mortality. In addition to fluids and vasopressors, the pre-hospital activation of an intra-hospital standardized hemorrhage control response including a transfusion protocol is recommended [34, 35]. A third factor that could explain the drop in SBP during the pre-hospital phase in patients with TBI and admission hypotension is the induction of anesthesia and intubation on scene. Yet, the difference in the rate of pre-hospital intubation between TBI patients with and without admission hypotension was not different. Also, when comparing TBI patients with admission hypotension who were intubated on scene with TBI patients with admission hypotension who were not intubated on scene, no difference in SBP on admission was found.

We found that more than one third of patients in group 1 and 2 were hypothermic (body temperature < 35 °C) on hospital arrival while the rate of admission hypothermia was low in patients without TBI (group 3). This difference could be caused by the higher rate of anesthesia in group 1 and 2 compared to group 3. General anesthetics greatly impair thermoregulation, reducing the thresholds for vasoconstriction and shivering [36], increasing the risk of hypothermia. Hypothermia is an independent predictor of mortality in trauma [37, 38] and hypothermia prevention is paramount, although often disregarded during the initial resuscitation and particularly difficult in the harsh environment encountered in mountain rescue.

We sought factors easily obtainable on scene that could predict admission hypotension in order to identify at risk patients on scene and eventually adopt a more aggressive treatment. We used a relatively novel method in the field of medicine, i.e. classification trees [13]. No single variable or any set of variables easily obtainable in the pre-hospital setting were able to predict admission hypotension with a reasonable sensitivity and specificity. Apart from GCS, the ISS and the AIS abdomen and AIS extremities were the variables most frequently selected in the classification trees. Both, the abdomen and the extremities can be a source of major bleeding, which affirms that hemorrhage is probably the main cause of hypotension multiple trauma patients with TBI and aggressive treatment of bleeding is paramount.

### Limitations

First, the number of cases collected in the IATR is limited when compared to the large data sets of urban trauma cases. Second, some data were missing, which is a limitation inherent to almost all registries. Both limitations curb the possibility to draw specific treatment protocols for mountain trauma causalities from our analysis. This highlights the need to continue to prospectively collect high quality data on trauma patients in mountain areas to perform further in-depth analyses using a larger sample size. Third, in the IATR, head and neck injuries are grouped in a single category (head/neck) and therefore the rate of isolated or concomitant spinal cord injuries, which could contribute to hypotension and hypothermia, is unknown. Fourth, we used SBP to define hypotension while the mean arterial pressure is more relevant for the cerebral perfusion and should be considered when available. Yet, SBP is much easier to obtain in the pre-hospital setting and the latest Brain Trauma Foundation guidelines recommend SBP target values [2].

## Conclusion

Multiple trauma in the mountains goes along with severe TBI in almost 50%. One third of patients with TBI is hypotensive on hospital arrival and this is associated with increased mortality. No single variable or set of variables easily obtainable at scene was able to predict admission hypotension in TBI patients. Hypothermia is frequent and hypothermia assessment and prevention therefore paramount for multiple trauma patients with TBI in mountain areas. Whether more aggressive pre-hospital volume resuscitation or vasopressor administration can improve survival in these patients needs to be investigated in further studies.

## Supplementary Information

**Additional file 1: Supplemental Table 1.** Variables selected, sensitivity and specificity of each classification tree.

## Data Availability

The datasets used and/or analysed during the current study are available from the corresponding author on reasonable request.

## Abbreviations

AIS: Abbreviated injury scale
aPTT: Activated Partial Thromboplastin Time
ATLS: Advance Trauma Life Support
BE: Base excess
GCS: Glasgow Coma Scale
IATR: International alpine trauma registry
INR: International normalized ration
ISS: Injury severity score
SBP: Systolic blood pressure
TBI: Traumatic brain injury

## References

[1] Majdan M, Plancikova D, Brazinova A, Rusnak M, Nieboer D, Feigin V, et al. Epidemiology of traumatic brain injuries in Europe: a cross-sectional analysis. Lancet Public Health. 2016;1(2):e76–83. 10.1016/S2468-2667(16)30017-2. 29253420 10.1016/S2468-2667(16)30017-2

[2] Carney N, Totten AM, O'Reilly C, Ullman JS, Hawryluk GW, Bell MJ, et al. Guidelines for the Management of Severe Traumatic Brain Injury, Fourth Edition. Neurosurgery. 2017;80(1):6–15. 10.1227/NEU.0000000000001432. 27654000 10.1227/NEU.0000000000001432

[3] Barton CW, Hemphill JC, Morabito D, Manley G. A novel method of evaluating the impact of secondary brain insults on functional outcomes in traumatic brain-injured patients. Acad Emerg Med. 2005;12(1):1–6. 10.1197/j.aem.2004.08.043. 15635130 10.1197/j.aem.2004.08.043

[4] Manley G, Knudson MM, Morabito D, Damron S, Erickson V, Pitts L. Hypotension, hypoxia, and head injury: frequency, duration, and consequences. Arch Surg. 2001;136(10):1118–23. 10.1001/archsurg.136.10.1118. 11585502 10.1001/archsurg.136.10.1118

[5] Krishnamoorthy V, Vavilala MS, Mills B, Rowhani-Rahbar A. Demographic and clinical risk factors associated with hospital mortality after isolated severe traumatic brain injury: a cohort study. J Intensive Care. 2015;3(1):46. 10.1186/s40560-015-0113-4. 26561524 10.1186/s40560-015-0113-4 PMC4641350

[6] Tohme S, Delhumeau C, Zuercher M, Haller G, Walder B. Prehospital risk factors of mortality and impaired consciousness after severe traumatic brain injury: an epidemiological study. Scand J Trauma Resusc Emerg Med. 2014;22(1):1. https://jintensivecare.biomedcentral.com/articles/10.1186/s40560-015-0113-4#citeas. 10.1186/1757-7241-22-1 PMC3892077 24393519

[7] Berry C, Ley EJ, Bukur M, Malinoski D, Margulies DR, Mirocha J, et al. Redefining hypotension in traumatic brain injury. Injury. 2012;43(11):1833–7. 10.1016/j.injury.2011.08.014. 21939970 10.1016/j.injury.2011.08.014

[8] Ausserer J, Moritz E, Stroehle M, Brugger H, Strapazzon G, Rauch S, et al. Physician staffed helicopter emergency medical systems can provide advanced trauma life support in mountainous and remote areas. Injury. 2017;48(1):20–5. 10.1016/j.injury.2016.09.005. 27650943 10.1016/j.injury.2016.09.005

[9] Rauch S, Dal Cappello T, Strapazzon G, Palma M, Bonsante F, Gruber E, et al. Pre-hospital times and clinical characteristics of severe trauma patients: a comparison between mountain and urban/suburban areas. Am J Emerg Med. 2018;36(10):1749–53. 10.1016/j.ajem.2018.01.068. 29395773 10.1016/j.ajem.2018.01.068

[10] Pietsch U, Strapazzon G, Ambühl D, Lischke V, Rauch S, Knapp J. Challenges of helicopter mountain rescue missions by human external cargo: need for physicians onsite and comprehensive training. Scand J Trauma Resusc Emerg Med. 2019;27(1):17. 10.1186/s13049-019-0598-2 PMC6374883 30760298

[11] Strapazzon G, Costanzi E, Bonsante F, Rilk C, Mair P, Brugger H. International Alpine trauma registry: preliminary results for trauma life support in the mountains. Resuscitation. 2013;84:S96–S7. 10.1016/j.resuscitation.2013.08.245.

[12] Dick WF, Baskett PJ. Recommendations for uniform reporting of data following major trauma--the Utstein style. A report of a working party of the international trauma Anaesthesia and critical care society (ITACCS). Resuscitation. 1999;42(2):81–100. 10.1016/S0300-9572(99)00102-1. 10617327 10.1016/s0300-9572(99)00102-1

[13] Krzywinski M, Altman N. Classification and regression trees. Nat Methods. 2017;14(8):757–8. 10.1038/nmeth.4370.

[14] Team RC. R: a language and environment for statistical computing. Vienna: R Foundation for Statistical Computing; 2017.

[15] Therneau T, Atkinson B, Ripley B. rpart: Recursive Partitioning and Regression Trees. R package version 4.1-11 ed, 2017. Available from: http://www.R-project.org/. Accessed 15 Oct 2020.

[16] Thole RT. Preparation and medical management of events in mountain and high-altitude environments. Curr Sports Med Rep. 2004;3(3):128–33. 10.1249/00149619-200406000-00004. 15122978 10.1249/00149619-200406000-00004

[17] Marsigny B, Lecoq-Jammes F, Cauchy E. Medical mountain rescue in the Mont-Blanc massif. Wilderness Environ Med. 1999;10(3):152–6. 10.1580/1080-6032(1999)010[0152:MMRITM]2.3.CO;2. 10560308 10.1580/1080-6032(1999)010[0152:mmritm]2.3.co;2

[18] Gosteli G, Yersin B, Mabire C, Pasquier M, Albrecht R, Carron PN. Retrospective analysis of 616 air-rescue trauma cases related to the practice of extreme sports. Injury. 2016;47(7):1414–20. 10.1016/j.injury.2016.03.025. 27206845 10.1016/j.injury.2016.03.025

[19] Timm A, Maegele M, Lefering R, Wendt K, Wyen H, TraumaRegister DGU. Pre-hospital rescue times and actions in severe trauma. A comparison between two trauma systems: Germany and the Netherlands. Injury. 2014;45(Suppl 3):S43–52. 25284234 10.1016/j.injury.2014.08.017

[20] Kudo D, Yoshida Y, Kushimoto S. Permissive hypotension/hypotensive resuscitation and restricted/controlled resuscitation in patients with severe trauma. J Intensive Care. 2017;5(1):11. 10.1186/s40560-016-0202-z. 34798698 10.1186/s40560-016-0202-z PMC8600688

[21] Beekley AC. Damage control resuscitation: a sensible approach to the exsanguinating surgical patient. Crit Care Med. 2008;36(7 Suppl):S267–74. 10.1097/CCM.0b013e31817da7dc. 18594252 10.1097/CCM.0b013e31817da7dc

[22] Shackford SR. Prehospital fluid resuscitation of known or suspected traumatic brain injury. J Trauma. 2011;70(5 Suppl):S32–3. 10.1097/TA.0b013e31821a5858. 21841567 10.1097/TA.0b013e31821a5858

[23] Wald SL, Shackford SR, Fenwick J. The effect of secondary insults on mortality and long-term disability after severe head injury in a rural region without a trauma system. J Trauma. 1993;34(3):377–81; discussion 81-2. 10.1097/00005373-199303000-00012. 8483178 10.1097/00005373-199303000-00012

[24] Geeraedts LM Jr, Kaasjager HA, van Vugt AB, Frölke JP. Exsanguination in trauma: a review of diagnostics and treatment options. Injury. 2009;40(1):11–20. 10.1016/j.injury.2008.10.007. 19135193 10.1016/j.injury.2008.10.007

[25] Spahn DR, Bouillon B, Cerny V, Duranteau J, Filipescu D, Hunt BJ, et al. The European guideline on management of major bleeding and coagulopathy following trauma: fifth edition. Crit Care. 2019;23(1):98. 10.1186/s13054-019-2347-3. 30917843 10.1186/s13054-019-2347-3 PMC6436241

[26] Sumann G, Moens D, Brink B, Brodmann Maeder M, Greene M, Jacob M, et al. Multiple trauma management in mountain environments - a scoping review : Evidence based guidelines of the International Commission for Mountain Emergency Medicine (ICAR MedCom). Intended for physicians and other advanced life support personnel. Scand J Trauma Resusc Emerg Med. 2020;28(1):117. 33317595 10.1186/s13049-020-00790-1 PMC7737289

[27] Tran A, Yates J, Lau A, Lampron J, Matar M. Permissive hypotension versus conventional resuscitation strategies in adult trauma patients with hemorrhagic shock: a systematic review and meta-analysis of randomized controlled trials. J Trauma Acute Care Surg. 2018;84(5):802–8. 10.1097/TA.0000000000001816. 29370058 10.1097/TA.0000000000001816

[28] Gupta B, Garg N, Ramachandran R. Vasopressors: do they have any role in hemorrhagic shock? J Anaesthesiol Clin Pharmacol. 2017;33(1):3–8. 10.4103/0970-9185.202185. 28413267 10.4103/0970-9185.202185 PMC5374828

[29] Hylands M, Toma A, Beaudoin N, Frenette AJ, D'Aragon F, Belley-Côté É, et al. Early vasopressor use following traumatic injury: a systematic review. BMJ Open. 2017;7(11):e017559. 29151048 10.1136/bmjopen-2017-017559 PMC5701980

[30] Brenner M, Stein DM, Hu PF, Aarabi B, Sheth K, Scalea TM. Traditional systolic blood pressure targets underestimate hypotension-induced secondary brain injury. J Trauma Acute Care Surg. 2012;72(5):1135–9. 10.1097/TA.0b013e31824af90b. 22673237 10.1097/TA.0b013e31824af90b

[31] Lou X, Lu G, Zhao M, Jin P. Preoperative fluid management in traumatic shock: a retrospective study for identifying optimal therapy of fluid resuscitation for aged patients. Medicine (Baltimore). 2018;97(8):e9966. 10.1097/MD.0000000000009966. 29465593 10.1097/MD.0000000000009966 PMC5841965

[32] Cohn SM, McCarthy J, Stewart RM, Jonas RB, Dent DL, Michalek JE. Impact of low-dose vasopressin on trauma outcome: prospective randomized study. World J Surg. 2011;35(2):430–9. 10.1007/s00268-010-0875-8. 21161222 10.1007/s00268-010-0875-8

[33] Sims CA, Holena D, Kim P, Pascual J, Smith B, Martin N, et al. Effect of low-dose supplementation of arginine vasopressin on need for blood product transfusions in patients with trauma and hemorrhagic shock: a randomized clinical trial. JAMA Surg. 2019;154(11):994–1003. 10.1001/jamasurg.2019.2884. 31461138 10.1001/jamasurg.2019.2884 PMC6714462

[34] Glen J, Constanti M, Brohi K. Assessment and initial management of major trauma: summary of NICE guidance. Bmj. 2016;353:i3051. 27333868 10.1136/bmj.i3051

[35] Hamada SR, Rosa A, Gauss T, Desclefs JP, Raux M, Harrois A, et al. Development and validation of a pre-hospital "Red Flag" alert for activation of intra-hospital haemorrhage control response in blunt trauma. Crit Care. 2018;22(1):113. 29728151 10.1186/s13054-018-2026-9 PMC5935988

[36] Sessler DI. Perioperative thermoregulation and heat balance. Lancet. 2016;387(10038):2655–64. 10.1016/S0140-6736(15)00981-2. 26775126 10.1016/S0140-6736(15)00981-2

[37] Balvers K, Van der Horst M, Graumans M, Boer C, Binnekade JM, Goslings JC, et al. Hypothermia as a predictor for mortality in trauma patients at admittance to the intensive care unit. J Emerg Trauma Shock. 2016;9(3):97–102. 10.4103/0974-2700.185276. 27512330 10.4103/0974-2700.185276 PMC4960783

[38] Wang HE, Callaway CW, Peitzman AB, Tisherman SA. Admission hypothermia and outcome after major trauma. Crit Care Med. 2005;33(6):1296–301. 10.1097/01.CCM.0000165965.31895.80. 15942347 10.1097/01.ccm.0000165965.31895.80

