# Supplementary material for: Severe traumatic brain injury and hypotension is a frequent and lethal combination in multiple trauma patients in mountain areas – an analysis of the prospective international Alpine Trauma Registry
Source: Scand J Trauma Resusc Emerg Med. 2021 Apr 30;29:61. doi: 10.1186/s13049-021-00879-1 (PMC8086074; doi:10.1186/s13049-021-00879-1)
Supplement: Supplementary file 1 — Additional file 1: Supplemental Table 1. Variables selected, sensitivity and specificity of each classification tree. [file 13049_2021_879_MOESM1_ESM.docx]

**Supplemental table 1.** Variables selected, sensitivity and specificity of each classification tree.

| **Variable** | **Classification tree** | | | | | | | | | |
| --- | --- | --- | --- | --- | --- | --- | --- | --- | --- | --- |
|  | **1** | **2** | **3** | **4** | **5** | **6** | **7** | **8** | **9** | **10** |
|  |  |  |  |  |  |  |  |  |  |  |
| AIS face | x | x |  |  |  |  |  |  |  |  |
| AIS thorax |  |  | x | x |  | x |  | x |  | x |
| AIS abdomen |  | x | x | x | x | x | x | x | x | x |
| AIS extremities | x | x | x | x |  |  | x | x |  | x |
| AIS external |  | x |  |  |  |  |  |  |  |  |
| ISS |  | x | x | x | x | x | x | x | x | x |
| SBP |  | x |  |  | x |  | x |  |  |  |
| GCS | x | x | x | x | x | x | x | x | x | x |
| age | x |  |  |  | x |  | x |  | x |  |
| quantity of crystalloid fluids | x |  |  |  |  |  | x |  |  |  |
| treatment free interval |  |  |  |  | x |  |  |  | x |  |
| gender |  |  |  |  |  |  |  | x |  |  |
|  |  |  |  |  |  |  |  |  |  |  |
| sensitivity (TBI with SBP < 110) | 17% | 21% | 50% | 70% | 42% | 7% | 58% | 20% | 36% | 63% |
| sensitivity (TBI with SBP ≥ 110) | 71% | 38% | 37% | 70% | 33% | 42% | 45% | 86% | 54% | 63% |
| specificity (no TBI) | 81% | 84% | 90% | 79% | 87% | 88% | 89% | 89% | 86% | 80% |
|  |  |  |  |  |  |  |  |  |  |  |

AIS, Abbreviated Injury Scale; GCS, Glasgow Coma Scale; ISS, Injury Severity Score; SBP, systolic blood pressure.
